# L-carnitine ameliorates the muscle wasting of cancer cachexia through the AKT/FOXO3a/MaFbx axis

**DOI:** 10.1186/s12986-021-00623-7

**Published:** 2021-11-01

**Authors:** Changpeng Wu, Mingxing Zhu, Zongliang Lu, Yaowen Zhang, Long Li, Na Li, Liangyu Yin, He Wang, Wei Song, Hongxia Xu

**Affiliations:** Department of Clinical Nutrition, Daping Hospital, Army Medical University (Third Military Medical University), Changjiangzhilu 10#, Chongqing, China

**Keywords:** Cancer cachexia, Muscle atrophy, L-carnitine, Colon-26, C2C12 cells, AKT, p70S6K, FOXO3a

## Abstract

**Background:**

Recent studies suggest potential benefits of applying L-carnitine in the treatment of cancer cachexia, but the precise mechanisms underlying these benefits remain unknown. This study was conducted to determine the mechanism by which L-carnitine reduces cancer cachexia.

**Methods:**

C2C12 cells were differentiated into myotubes by growing them in DMEM for 24 h (hrs) and then changing the media to DMEM supplemented with 2% horse serum. Differentiated myotubes were treated for 2 h with TNF-α to establish a muscle atrophy cell model. After treated with L-carnitine, protein expression of MuRF1, MaFbx, FOXO3, p-FOXO3a, Akt, p-Akt, p70S6K and p-p70S6K was determined by Western blotting. Then siRNA-Akt was used to determine that L-carnitine ameliorated cancer cachexia via the Akt/FOXO3/MaFbx. In vivo, the cancer cachexia model was established by subcutaneously transplanting CT26 cells into the left flanks of the BALB/c nude mice. After treated with L-carnitine, serum levels of IL-1, IL-6 and TNF-α, and the skeletal muscle content of MuRF1, MaFbx, FOXO3, p-FOXO3a, Akt, p-Akt, p70S6K and p-p70S6K were measured.

**Results:**

L-carnitine increased the gastrocnemius muscle (GM) weight in the CT26-bearing cachexia mouse model and the cross-sectional fiber area of the GM and myotube diameters of C2C12 cells treated with TNF-α. Additionally, L-carnitine reduced the protein expression of MuRF1, MaFbx and FOXO3a, and increased the p-FOXO3a level in vivo and in vitro. Inhibition of Akt, upstream of FOXO3a, reversed the effects of L-carnitine on the FOXO3a/MaFbx pathway and myotube diameters, without affecting FOXO3a/MuRF-1. In addition to regulating the ubiquitination of muscle proteins, L-carnitine also increased the levels of p-p70S6K and p70S6K, which are involved in protein synthesis. Akt inhibition did not reverse the effects of L-carnitine on p70S6K and p-p70S6K. Hence, L-carnitine ameliorated cancer cachexia via the Akt/FOXO3/MaFbx and p70S6K pathways. Moreover, L-carnitine reduced the serum levels of IL-1 and IL-6, factors known to induce cancer cachexia. However, there were minimal effects on TNF-α, another inducer of cachexia, in the in vivo model.

**Conclusion:**

These results revealed a novel mechanism by which L-carnitine protects muscle cells and reduces inflammation related to cancer cachexia.

## Introduction

The incidence and mortality of cancer have been increasing for several decades [1]. Based on the data from population-based registries now available through the Office for Cancer Registry of the National Cancer Center China, about 3,929,000 new cancer cases and 2,388,000 cancer-related deaths were reported in China in 2015 [2]. Patients with advanced cancer often show symptoms of cancer cachexia, which is characterized by a loss of skeletal muscle and leading to significant weight loss [3]. Cancer cachexia will lead to a reduced tolerance of cancer treatment, a poorer quality of life and decreased survival [3, 4]. Up to 80% of cancer patients have cachexia, and it has been estimated that more than 30% of cancer patients die due to cachexia [5, 6].

Muscle wasting is the core feature of cancer cachexia [3], which is mainly due to the reduction of muscle protein synthesis and/or acceleration of protein degradation [7]. Cancer patients produce many inflammatory cytokines [8, 9], such as tumor necrosis factor-alpha (TNF-α) [10, 11], proteolysis-inducing factor (PIF), interleukin-1 (IL-1) [9, 12], and interleukin-6 (IL-6) [8, 13]. These cytokines act directly on skeletal muscle cells and activate multiple signaling pathways within the cell, further promoting protein degradation via the proteasome pathway and a calcium-dependent pathway [10, 11, 14]. Studies have shown that muscle RING-type E3 ubiquitin ligase 1 (MuRF1, TRIM63) and muscle atrophy F-box protein (MaFbx) in the ubiquitin–proteasome pathway are closely related to the degradation of muscle protein in cachexia patients [15–17].

In atrophying muscles, there is activation of the Forkhead box O transcription factor family (FOXO), as well as NF-κB, which can be activated by TNF-α, soluble TNF-like weak inducer of apoptosis (TWEAK), or IL-1 [15, 16]. FOXO3a itself induces a set of atrophy-related genes, specifically the muscle-specific ubiquitin ligases, MAFbx and MuRF-1, which promote the breakdown of the myofibrillar apparatus [17]. PI3K/AKT signaling is the most prominent pathway that leads to FOXO3a phosphorylation [18], which results in its subsequent ubiquitination by MDM2 [19]. PI3K/AKT signaling also leads to the activation of mammalian target of rapamycin (mTOR), and p70S6, and is known to promote muscle synthesis [20].

As the etiology of cancer cachexia is multifactorial and complex, the deterioration resulting in muscle wasting can not be fully reversed by conventional nutritional support [3]. L-carnitine is an amino acid whose main function is to promote the β-oxidation of long-chain fatty acids and to produce energy. It is often used in the treatment of diseases affecting the cardiovascular system, nervous system, liver, and kidney [21, 22]. A few studies showed that oral supplementation of L-carnitine exerts a beneficial effect against cancer cachexia [23, 24]. The present study was performed to confirm whether L-carnitine has beneficial effects against cancer cachexia and to uncover a possible mechanism of action using both in vitro and in vivo studies.

## Materials and methods

### Chemicals and reagents

L-carnitine was obtained from Sigma Aldrich (St. Louis, MO, USA). The anti-AKT (1:1000, ab227385), anti-phospho-AKT1 (Ser 473) (1:1000, ab81283), anti-FOXO3a (1:1000, ab12162), and anti-phospho-FOXO3a (Ser 253) (1:1000, ab154768) antibodies were purchased from Abcam Corp. (Cambridge, MA, USA). Anti-p70S6K (1:1000, 2217 s) and anti-phospho-p70S6K (Thr 389) (1:1000, 4858 s) were purchased from Cell Signaling Technology (Danvers, MA, USA). Anti-MAFbx (1:1000, EB09088) was purchased from Everest Biotech. (Shanghai, China). Anti-MuRF1 (1:1000, GTX110475) was purchased from GeneTex (Irvine, CA, USA). The anti-GAPDH (1:1000, TA309157), anti-mouse and anti-rabbit secondary antibodies (1:5000–1:1000) were purchased from ZSGB-BIO (Beijing, China). Goat anti-mouse/rabbit IgG horseradish peroxidase-conjugated antibodies were purchased from Bio-Rad (Hercules, CA, USA). The trypsin and BCA assay kit were purchased from Beyotime (Shanghai, China). Lipofectamine™ 2000 was purchased from Invitrogen (Carlsbad, CA, USA) and OPTI-MEM Reduced Serum Medium were purchased from Gibco (Langley, USA). The ELISA kit was purchased from Abebio (Wuhan, China).

### In vitro cachexia model

**Fig. 1:**
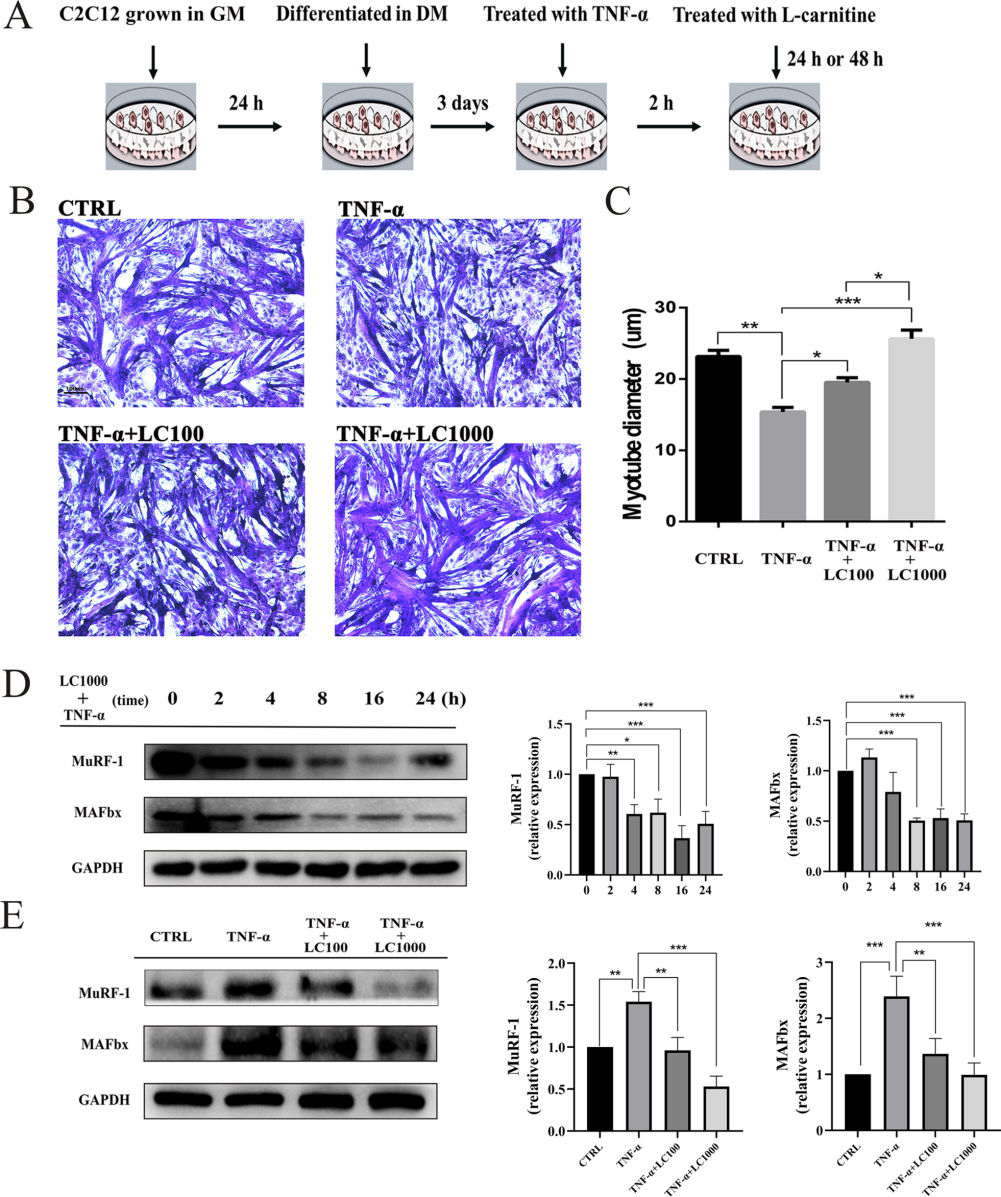
L-carnitine blocks TNF-α-induced myotube atrophy in C2C12 cells. **A** C2C12 cells were grown in DMEM (growth medium, GM) for 24 h and then changed to differentiation medium (DM) for 3 days. After that, differentiated C2C12 cells were treated for 2 h with 100 ng/ml TNF-α, and then were simultaneously treated with L-carnitine and TNF-α for an additional 24 or 48 h. **B** Photomicrographs of cultured myotubes after treatment with water (as vehicle), 100 ng/ml TNF-α, a combination of TNF-α (100 ng/ml) with 100 μg/ml or TNF-α (100 ng/ml) with 1000 μg/ml of L-carnitine. **C** Quantification of the average myotube diameter after 24 h of treatment with water, TNF-α, or TNF-α in combination with the two different concentrations of L-carnitine. Data were plotted as the means (n ≥ 90 myotubes per condition) ± SEM. **D** A representative Western blot of MuRF1, MAFbx and GADPH following treatment with the combination of 100 ng/ml TNF-α and 1000 μg/ml of L-carnitine after different time points. A representative Western blot of MuRF1, MAFbx and GADPH protein expression. **E** A representative Western blot of MuRF1, MAFbx and GADPH protein expression. The data shown represent the means ± SEM of three independent experiments. *P < 0.05, **P < 0.01, ***P < 0.001

The TNF-α-induced C2C12 model is a widely used muscle cell atrophy model that simulates the muscle wasting of cancer cachexia [10, 11, 25]. The C2C12 mouse myoblast cell line was purchased from the Chinese Academy of Sciences/Stem Cell Bank (Shanghai, China). C2C12 cells were differentiated into myotubes by growing them in DMEM for 24 h and then changing the media to DMEM supplemented with 2% horse serum,1 mM sodium pyruvate, and 10 mM HEPES for 3 days [26, 27]. Differentiated myotubes were treated for 2 h with 100 ng/ml TNF-α (PeproTech, Rocky Hill, USA) to establish a muscle atrophy cell model. L-carnitine treatments were carried out simultaneously with the TNF-α treatment (100 μg/ml L-carnitine; TNF-ɑ + LC100 group or 1000 μg/ml L-carnitine; TNF-ɑ + LC1000 group) for an additional 24 h or 48 h (Fig. 1A).

### Assessment of cell differentiation

The differentiated C2C12 cells were stained with Crystal Violet Staining Solution (Beyotime, China), and images were taken using an inverted microscope (Nikon ECLIPSE Ti, Japan). The myotube diameters (μm) were measured at ¼, ½, and ¾ along the length of a selected myotube and the average diameters were calculated. A total of 30 myotubes were measured for each group in each single experiment. Three separate experiments were performed to determine the myotube diameters.

### Transfection of siRNAs

C2C12 cells were treated with 100 ng/mL TNF-α for 2 h to establish the muscle atrophy cell model. The cells were transfected with 3 μg of AKT-siRNA (RIBOBIO, China) in 240 μL OPTI-MEM Reduced Serum Medium for 5–7 h using a previously reported procedure. The cells were then switched to DMEM differentiation medium and treated with 1000 μg/mL of L-carnitine for an additional 24 h. Thereafter, the cells were harvested and Western blotting was performed for p-p70S6K, p70S6K, p-FOXO3a, FOXO3a, MuRF-1, MAFbx and GAPDH. The siRNA sequences used were as follows: siRNA-AKT#1 (control): GCACCTTTATTGGCTACAA; siRNA-AKT#1:CCATGAACGAGTTTGAGTA;siRNA-AKT#2:GTGCCACTATGAAGACATT.

### Western blot analyses

For the in vitro studies, differentiated C2C12 cells were collected at the end of experiments by cell lysis in ice-cold RIPA buffer. The lysates were subjected to SDS-PAGE according to a previously reported procedure [28]. The PVDF membranes were incubated with the appropriate primary antibodies overnight at 4 °C with gentle shaking, and then incubated with a goat anti-mouse/rabbit IgG horseradish peroxidase-conjugated antibody. Conjugated proteins were detected by an enhanced chemiluminescent immunoblotting detection system, the ChemiDoc™ Touch Imaging System (BIO RAD, CA, USA) was performed. The gastrocnemius muscle tissues from mice were homogenized and solubilized in RIPA lysis buffer (Beyotime, China). The resulting suspension was separated by centrifugation at 12,000 rpm for 30 min at 4 °C, and the protein concentrations was performed as in a previous report [28]. Proteins were subsequently electrophoretically separated and transferred onto PVDF membranes for Western blotting as described above. Image J software was used to perform a densitometric analysis of the blots. The levels of phosphorylated and total proteins were normalized to the expression of GAPDH. Finally, the ratio between phosphorylated and total protein was determined.

### Animals and cachexia model

The mouse colon cancer cell line, CT26, which shows high tumorigenicity and low metastatic potential [29], was implanted into mice to generate an in vivo cancer cachexia model [8, 29, 30]. CT26 cells were purchased from the Chinese Academy of Sciences/Stem Cell Bank (Shanghai, China). Cells were seeded on Falcon dishes at 37 °C with 5% CO_2_ in Dulbecco’s modified Eagle medium (DMEM) supplemented with 10% heat-inactivated fetal bovine serum (FBS), 100 U/ml penicillin (Beyotime, China) and 100 mg/ml streptomycin (Beyotime, China) [26]. BALB/c nude mice (6 weeks old) were purchased from the Laboratory Animal Center of Army Medical University (AMU) (Chongqing, China) and maintained at a constant temperature and humidity, with free access to food and water. All the animals were handled in compliance with the Policy on the Humane Care and Use of Laboratory Animals. The experimental protocol was approved by the Animal Care Committee of AMU in accordance with the government’s guidelines for animal manipulations in China. The cancer cachexia model was established by subcutaneously transplanting 5 × 10^6^ CT26 cells into the left flanks of male mice as described in a previous study [31] (as day 0, D0). The tumors became palpable on day 6 (D6). After 18 days (D24) of L-carnitine intervention, the four groups of mice were sacrificed using 4% isoflurane, then blood and tissue samples were collected and evaluated.

### Treatment of cachexic mice with L-carnitine

L-carnitine was dissolved in 0.9% sodium chloride (NaCl). A total of 30 male BALB/c nude mice were randomly divided into four groups: a CT26 + 0.9%NaCl group (8 mice bearing CT26 tumors, treated with vehicle, as a control model of cancer cachexia), a 0.9%NaCl group (6 mice without tumors, treated with the vehicle, as normal controls), a CT26 + LC1 group (8 mice with CT26 tumors, treated with1 mg/kg bodyweight L-carnitine) and a CT26 + LC10 group (8 mice with CT26 tumors treated with10 mg/kg bodyweight L-carnitine). All of the treatments (0.9%NaCl or different doses of L-carnitine) were administered by oral gavage daily from Day 6 (D6) to Day 24 (D24) (Fig. 4A).

The body weight, food intake, and tumor size were measured every other day. Tumor length and width were measured using digital calipers, then the tumor size (volume) was calculated using the formula: 0.5 × tumor width (cm) × length (cm)^2^. At the end of the experiments, all of the mice were sacrificed following an overdose of inhalation anesthesia with isoflurane. While mice were still alive but anesthetized, blood was collected from the supraorbital veins for the assessment of biochemical parameters. Tumors were also dissected and weighed. The true body weight was calculated by subtracting the tumor weight from the total body weight. The gastrocnemius muscles of both hind legs were stripped, weighed and then used for pathological examinations and molecular biological analyses.

### Histopathology of the gastrocnemius muscle and tumor

The gastrocnemius muscles were fixed with 4% paraformaldehyde in 0.2 mol/LPBS, washed with PBS, and stained with hematoxylin and eosin (HE) solution. Images of muscle sections were recorded using the Image J software.

### Serum cytokine assays

The serum IL-1, IL-6 and TNF-α levels were detected by ELISA (Abebio, Wuhan, China) following the manufacturer’s instructions. The blood glucose, triglyceride and total cholesterol levels were measured using a biochemical analyzer (UniCelDxC 800 Synchron, USA) in the clinical laboratory of our hospital.

### Statistical analysis

All data were expressed as the means ± SD and were analyzed using an analysis of variance (ANOVA) followed by Tukey’s test (*P ≤ 0.05, **P ≤ 0.01, ***P ≤ 0.001) for post-hoc comparisons. All tests were two-sided and *P* ≤ 0.05 was considered statistically significant.

## Results

### L-carnitine blocks TNF-α-induced myotube atrophy in C2C12 cells

The TNF-α-treated C2C12 cells model is an established in vitro model of muscle atrophy [10]. Figure 1B shows that, as expected, the myotube differentiation process was decreased following exposure to 100 ng/ml of TNF-α for 2 h. The myotubes were slimmer, and their growth was attenuated and disordered.

Following this 2 h of TNF-α treatment, the myotube cells were treated with 100 μg/ml or 1000 μg/ml of L-carnitine for 48 h. The L-carnitine treatment resulted in a hypertrophic appearance (Fig. 1B) and an increase in the myotube diameters (Fig. 1C), with the exposure to 1000 μg/mL of L-carnitine resulting in stronger effects compared with 100 μg/mL of L-carnitine.

Two ubiquitin ligases, MAFbx and MuRF1, have previously been shown to be rapidly induced in multiple models of muscle atrophy, including the TNF-α-induced muscle wasting model [14, 32]. We therefore checked the protein expression of MAFbx and MuRF1 in the TNF-α-induced myotube atrophy model and the CT26 tumor-bearing mice to confirm the anti-catabolic effects of L-carnitine.

After TNF-α treatment for 2 h, the MuRF1 and MAFbx protein expression levels were significantly elevated, and these levels were decreased in a time-dependent manner following the addition of L-carnitine treatment (Fig. 1D). We also examined the time course of the anti-catabolic effects of L-carnitine by checking the MAFbx and MuRF1 expression levels after exposure to different concentrations of TNF-α and L-carnitine. The MuRF1 and MAFbx protein levels were significantly increased by TNF-α treatment, but were decreased by L-carnitine treatment (Fig. 1E). The TNF-α-induced upregulation of MAFbx and MuRF1 was almost completely reversed by treatment with either 100 μg/ml or 1000 μg/ml of L-carnitine. These findings demonstrate that L-carnitine inhibits muscle atrophy and induces hypertrophy.

### L-carnitine activates the AKT pathway, decreases FOXO3a protein expression, promotes FOXO3a phosphorylation and suppresses E3 ubiquitin ligases in vitro

**Fig. 2:**
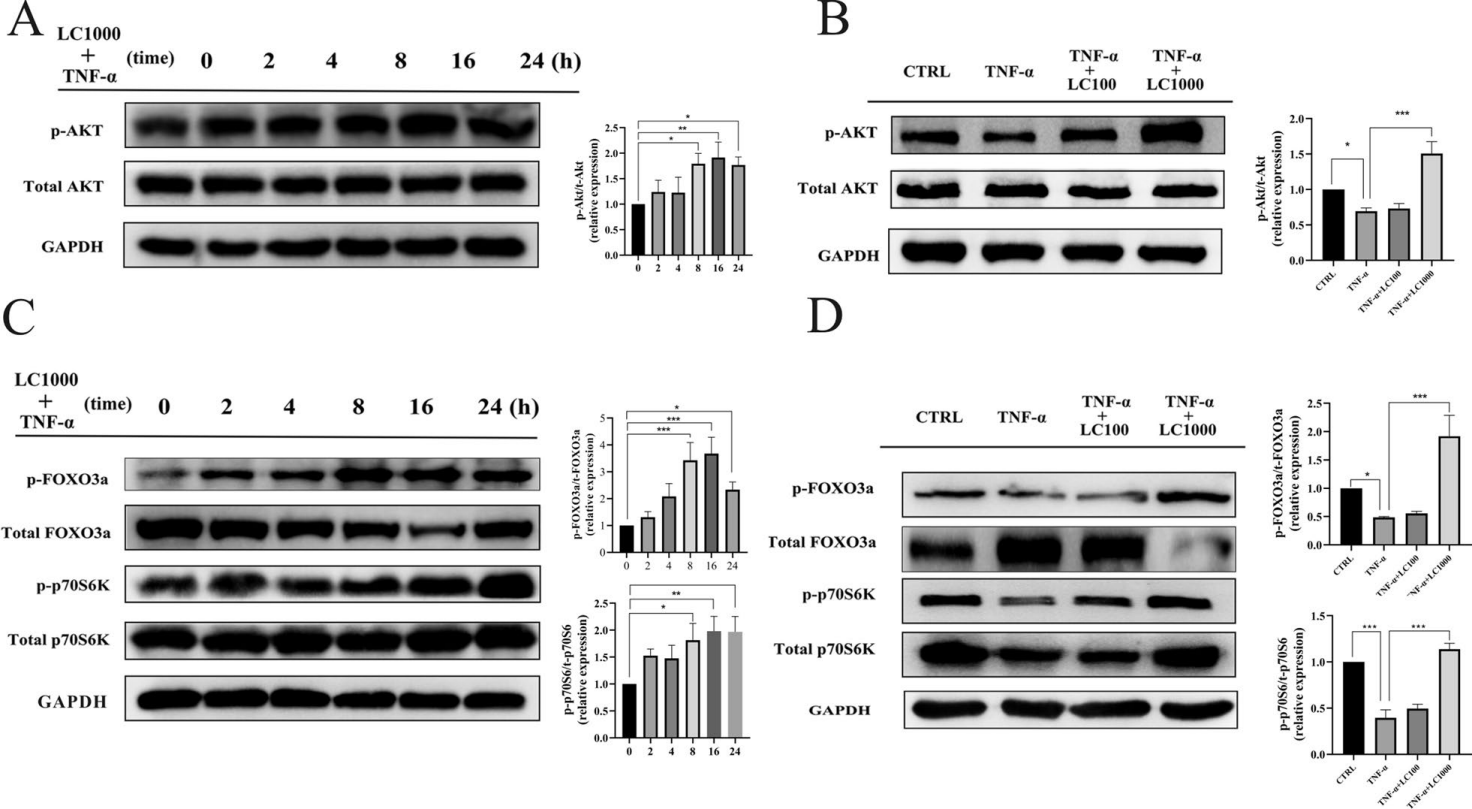
L-carnitine activates the AKT/FOXO3a pathway, increases p70S6k protein expression, and promotes p70S6k phosphorylation in vitro. **A** The expression levels of total AKT and p-AKT in the C2C12 myotubes were determined by Western blotting at different time points after 1000 μg/ml L-carnitine treatment (0, 2, 4, 8, 16, 24 h) following stimulation with TNF-α (100 ng/mL) for 2 h. **B** C2C12 myotubes were stimulated with TNF-α (100 ng/mL) for 2 h and then treated with different concentrations (100 or 1000 μg/mL) of L-carnitine for 16 h, and then the expression levels of total AKT and p-AKT were determined by Western blotting. **C** The expression levels of total p-p70S6K, p70S6K, p-FOXO3a and total FOXO3a were determined by Western blotting. **D** C2C12 myotubes were stimulated with TNF-α (100 ng/mL) for 2 h and then treated with different concentrations (100 or 1000 μg/mL) of L-carnitine for 16 h, and then the expression levels of total p-p70S6K, p70S6K, p-FOXO3a and total FOXO3a were determined by Western blotting

A previous study suggested that TNF-ɑ promotes muscle loss by inhibiting AKT. In the present in vitro study, we confirmed that the levels of p-Akt were increased by L-carnitine in a time-dependent manner in the myotubes with TNF-α-induced atrophy (Fig. 2A), and the levels reached the peak at 16 h. Therefore, we observed the dose effects of L-carnitine at 16 h. The data shown in Fig. 2B demonstrate that the p-Akt protein expression levels were significantly decreased by TNF-α treatment and then increased by 1000 ug/ml L-carnitine treatment, but 100 ug/ml L-carnitine showed no effect on p-Akt. It has been widely reported that a reduction of AKT phosphorylation leads to FOXO3a activation and increased MAFbx and MuRF1 transcription, thus controlling cell survival and cell proliferation. We therefore examined the protein expression of FOXO3a and p-FOXO3a in the in vitro model. As shown in Fig. 2C, the protein expression levels of FOXO3a were decreased and p-FOXO3a were increased by L-carnitine treatment in a time-dependent manner, but the FOXO3a protein expression level was increased at 24 h.

In addition to muscle degradation, Akt also promotes protein synthesis through p70S6K pathways. Our results demonstrated that the p70S6K and p-p70S6K levels were increased by L-carnitine treatment in a time-dependent manner (Fig. 2C). We also checked the dose-dependent expression of t-p70S6K, p-p70S6K, FOXO3a and p-FOXO3a following exposure to TNF-ɑ, with or without L-carnitine, and found that treatment with 1000 μg/L L-carnitine increased the protein expression levels of p-FOXO3a, t-p70S6K and p-p70S6K and decreased the level of FOXO3a (Fig. 2D).

**Fig. 3:**
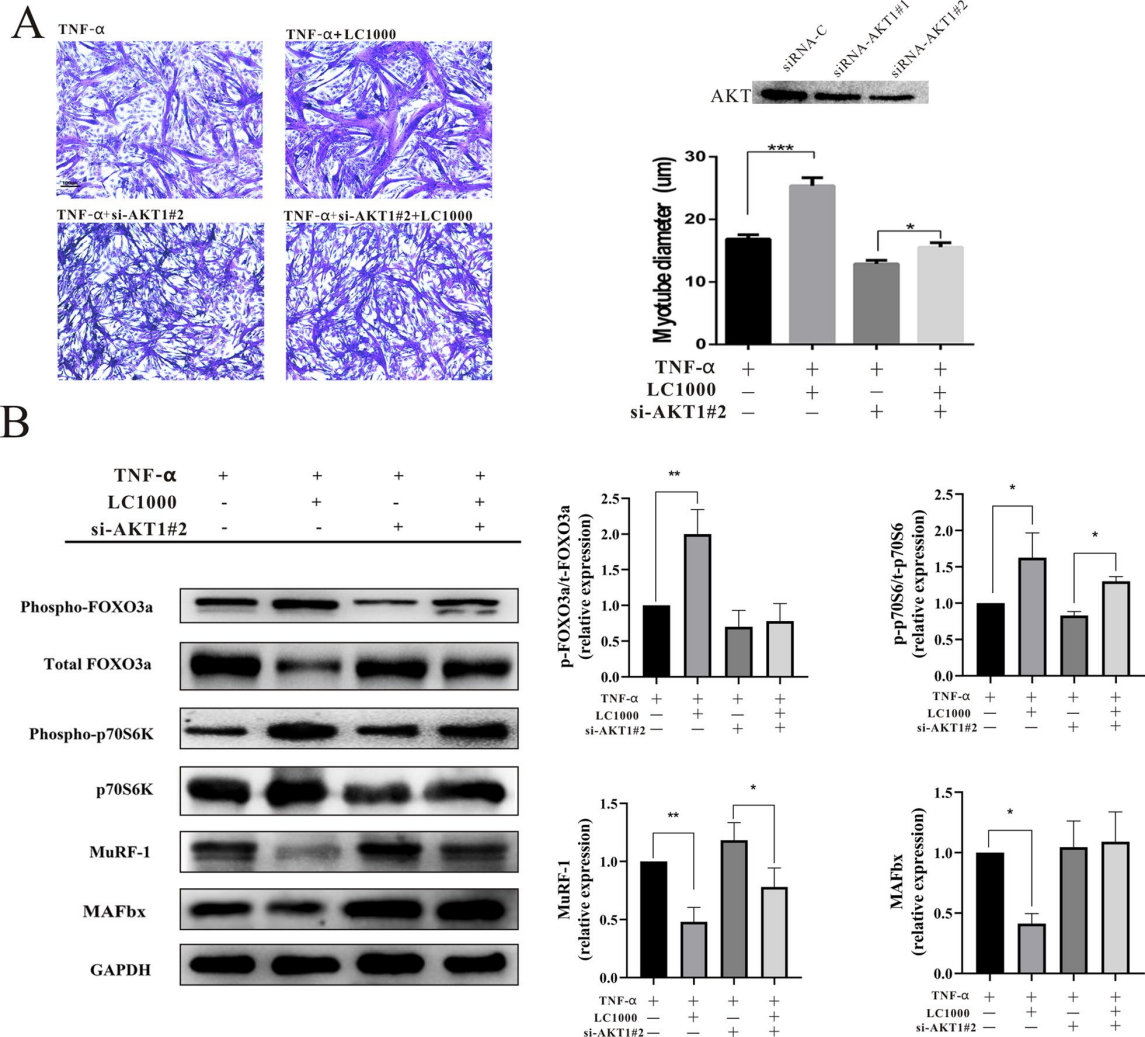
The effects of L-carnitine are mediated by activation of the AKT/FOXO3a/MAFbx pathway. **A** The expression levels of AKT in myotube cultures treated with 50 nM si-RNA AKT1 were determined by Western blotting. After being pre-treated with 50 nM si-RNA AKT1 #2 for 6 h, myotube cultures were treated with 1000 μg/mL L-carnitine or the vehicle control for 48 h, then photomicrographs were taken. The diameters of the C2C12 myotubes. **B** After being pre-treated with 50 nM si-RNA AKT1#2 for 6 h, C2C12 myotubes were treated with 1000 μg/mL L-carnitine or the vehicle control for 24 h, then the p70S6K, p-p70S6K, FOXO3a, p-FOXO3a, MAFbx and MuRF1 protein levels were determined by Western blotting. The data shown represent the means ± SEM of three independent experiments. *P < 0.05, **P < 0.01, ***P < 0.001

We then transfected cultured myotubes with siRNA against AKT or with non-targeting (scrambled) siRNA to test the effects of L-carnitine on TNF-α-induced FOXO3a, p70S6K, MAFbx and MuRF1 expression in the absence of AKT. Photomicrographs of myotube cultures showed the effective inhibition of AKT by transfection with the siRNA against AKT, which made the myotube diameters smaller (Fig. 3A). Transfection with the siRNA increased the TNF-α-induced FOXO3a and MAFbx expression levels while reducing the p-FOXO3a expression. It also had slight effects on the expression levels of p70S6K, p-p70S6K and MuRF1 (Fig. 3B).

Taken together, the present results support the idea that L-carnitine induces activation of the AKT pathway, and inhibits the TNF-α-induced increase in MAFbx expression by decreasing FOXO3a protein expression and promoting FOXO3a phosphorylation.

### L-carnitine ameliorates muscle mass atrophy and body weight loss

**Fig. 4:**
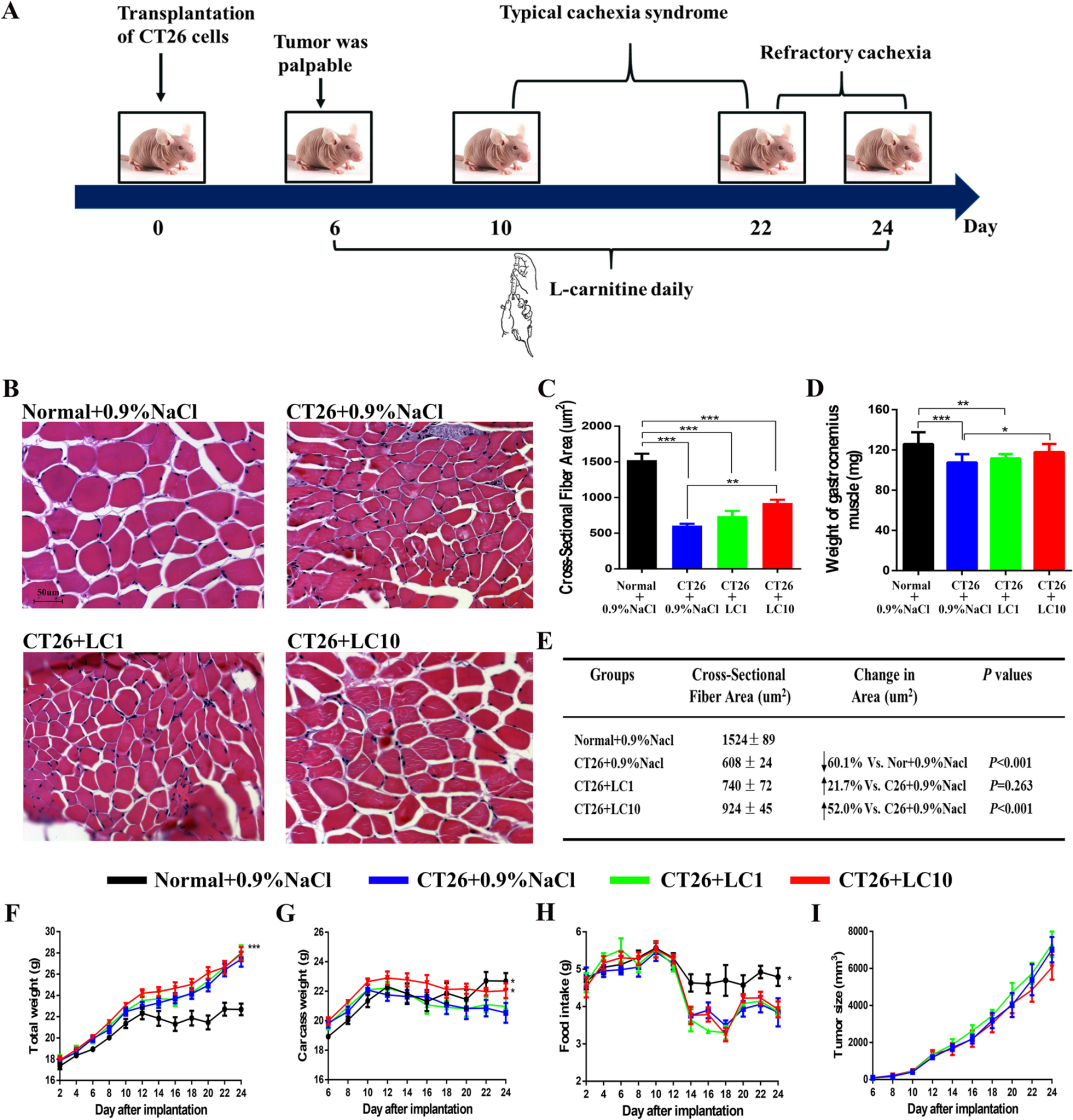
L-carnitine ameliorates muscle mass atrophy and body weight loss, but without affecting food intake or tumor growth in CT26 tumor-bearing mice. **A** CT26 cells were transplanted into BALB/c nude mice (on day 0, D0) and the tumors became palpable on day 6 (D6). Subsequently, L-carnitine was administered at a dose of 1 mg/kg or 10 mg/kg by oral gavage daily from D6 to Day 24 (D24). Gastrocnemius muscles (GM) were examined at the end of the experiments by **B** HE-staining, **C** determining the cross-sectional fiber area, **D** weighing the GM of both hind legs and **E** determining the differences in the cross-sectional fiber area of the GM between groups. The body weight, food intake and tumor size were measured from the beginning to the end of the experiment, as shown in (**F**). The total weight of mice, **G** carcass weight of the mice, **H** food intake and **I** tumor size are shown. Data were plotted as the means ± SEM. *P < 0.05, **P < 0.01, ***P < 0.001. Control = animals treated with the vehicle (0.9% NaCl; n = 6); other three groups (n = 8 for each)

Although our in vitro findings demonstrated a significant impact of L-carnitine on the TNF-α-induced C2C12 cell cachexia model and provided insight into the specific mechanism, drug metabolism in vivo is complex. We therefore used a well-established in vivo model of cachexia to assess whether L-carnitine would reduce the development of cachexia in mice. The HE-staining of the gastrocnemius muscle (GM) confirmed that the myofibers were thinner and less ordered (evidence of wasting) in the cachexia control (CT26 + 0.9% NaCl group) compared with the normal control group (Normal + 0.9%NaCl group), as noted in the cross-sections of muscle fibers. L-carnitine treatment ameliorated the muscle wasting, with the 10 mg/kg/d L-carnitine treatment nearly reversing the muscle fiber changes (Fig. 4B). The cross-sectional fiber area (CFA) of the GM showed that 10 mg/kg/d L-carnitine treatment resulted in a 52.0% increase in area compared to the cachexia control, while 1 mg/kg/d L-carnitine treatment resulted in a 21.7% increase (Figur 4C, E). The GM of both hind legs were weighed, and the data showed that 10 mg/kg/d L-carnitine treatment almost completely prevented the weight loss of the GM, with the muscles close in weight to the those of the normal control (Fig. 4D).

There was a progressive increase in body weight that stopped on the 10^th^ day, with slight fluctuations continuing in the normal control group. The increase in the total body weight of the cachexic mice and mice treated with L-carnitine (Fig. 4F) was mainly due to tumor growth (Fig. 4I). Due to the cachexia, the body weight of the cachexia control group began to decrease on the 10^th^ day. The weight in the 1 mg/kg/d L-carnitine (CT26 + 1 mg/kg LC) group began to decline on the 12^th^ day, and that in the 10 mg/kg/d L-carnitine (CT26 + 10 mg/kg LC) group began to decline on the 14^th^ day (Fig. 4G). Within 3 weeks after cancer cell implantation, the body weights (total weight minus estimated tumor weight) had decreased by > 5% in all of the cachexia groups.

The food intake also decreased in the tumor-bearing mice (Fig. 4H). At the end of the experiment, the food intake had decreased by about 20% in all three groups of tumor-bearing mice, which showed that low- or high-dose L-carnitine treatment did not improve the anorexia associated with cachexia.

### L-carnitine activates the AKT/FOXO3a/MaFbx pathway, increases p70S6k protein expression, and promotes p-p70S6k phosphorylation to ameliorate the muscle wasting of cancer cachexia in CT26-bearing mice

**Fig. 5:**
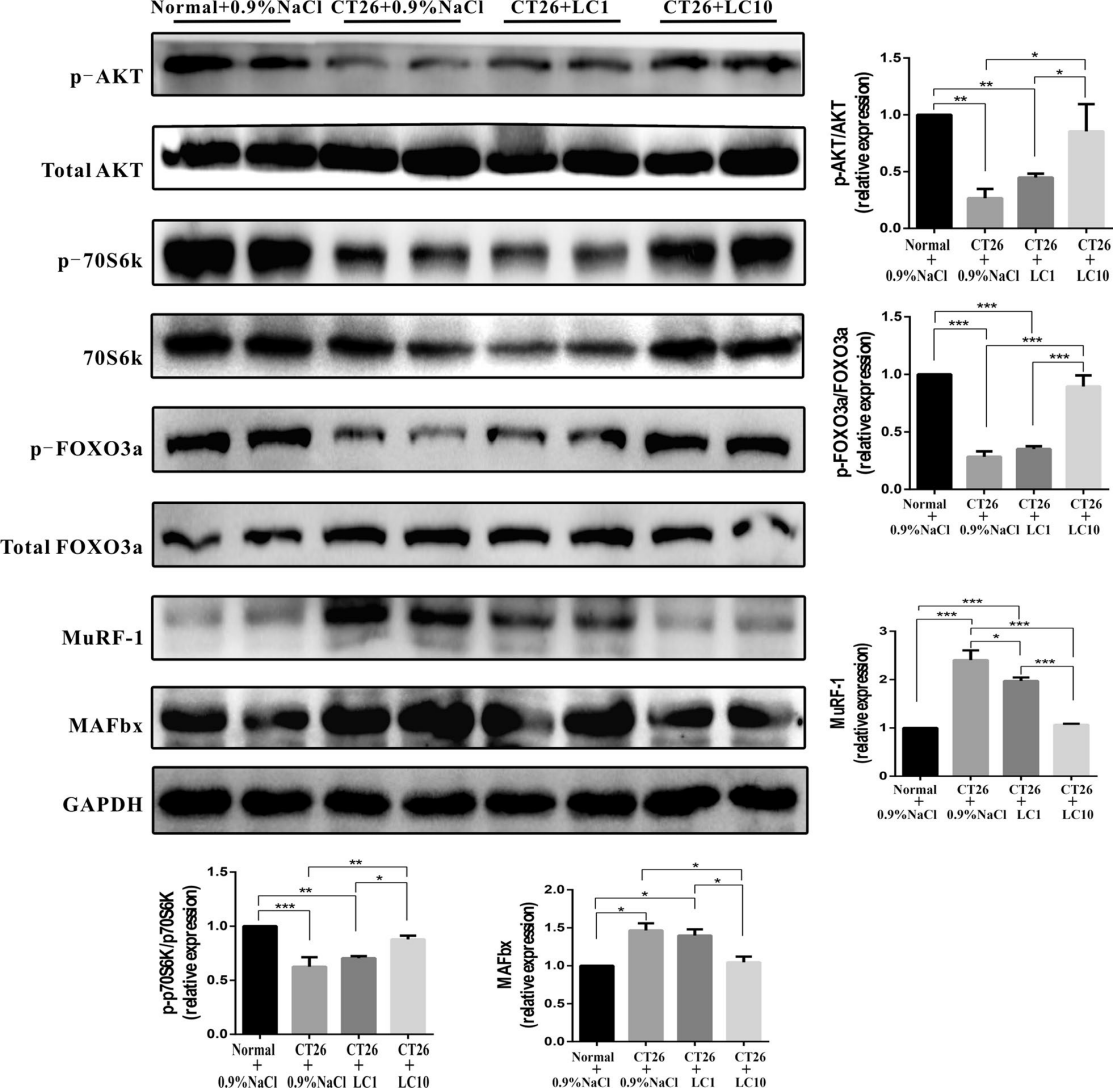
L-carnitine activates the AKT/FOXO3a/MaFbx pathway, increase p70S6k protein expression, and promotes p-p70S6k phosphorylation to ameliorates the muscle wasting of cancer cachexia in CT26-bearing mice. The expression levels of total AKT, p-AKT, p70S6K, p-p70S6K, total FOXO3a, p-FOXO3a, MuRF1 and MAFbx were determined by Western blotting tissue homogenates of the gastrocnemius muscle from mice with cancer cachexia

In our in vivo study, p-Akt and p-FOXO3a were decreased in CT26 tumor-bearing mice, and FOXO3a and MAFbx were increased, which could partly explain the muscle loss in the cachectic mice (Fig. 5). However, L-carnitine treatment reduced this effect and enhanced the activation of the AKT/FOXO3a/MaFbx axis.

The present data revealed that there was an increase in the p70S6k and p-p70S6k expression levels in the mice treated with 10 mg/kg/d L-carnitine compared to the levels in saline-treated cachectic mice (Fig. 5). A decreased MuRF1 level was associated with reduced muscle degradation, as shown in Fig. 5.

**Fig. 6:**
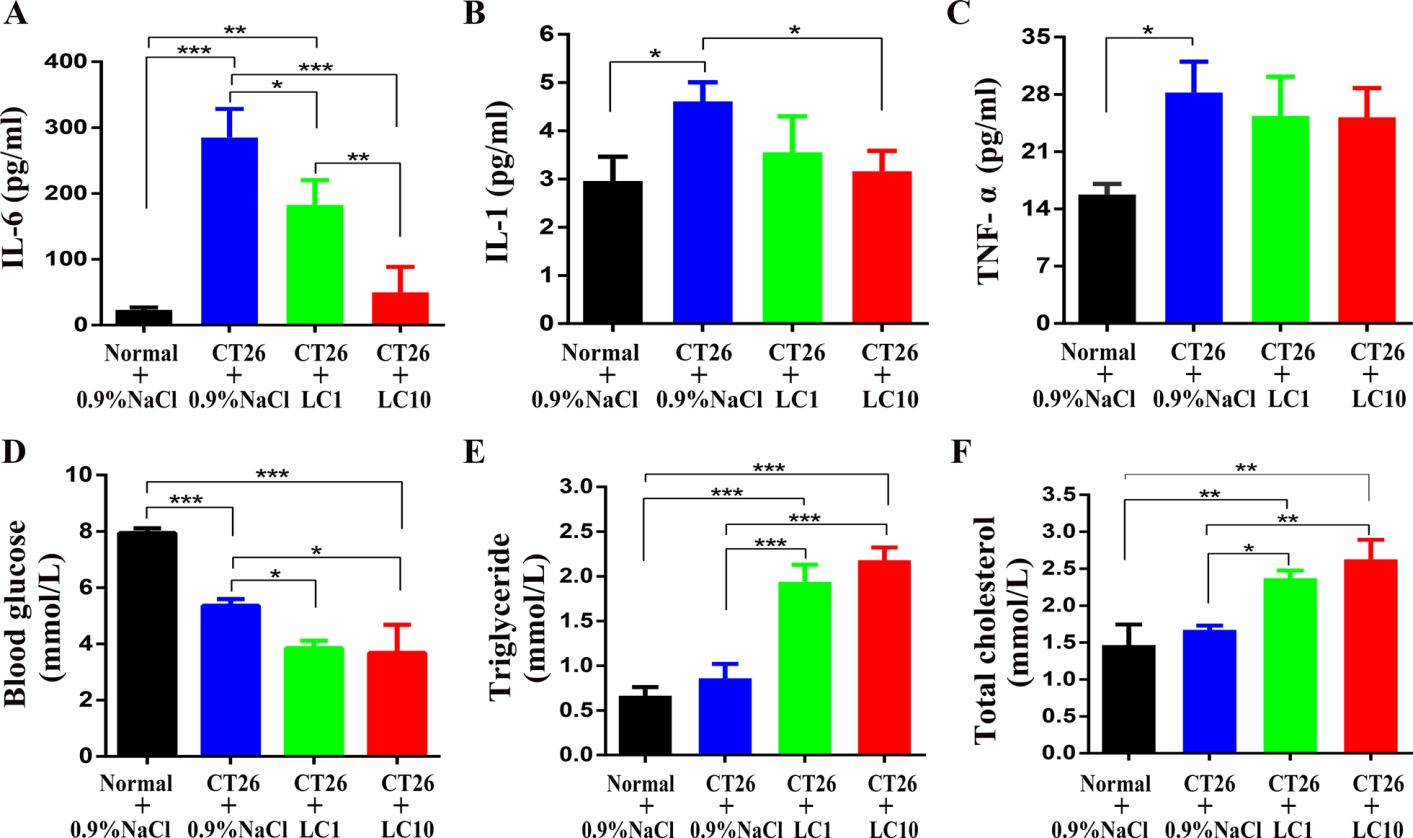
L-carnitine regulates the serum levels of inflammatory cytokines and affects glycolipid metabolism in CT26 tumor-bearing cachexic mice. At the end of the experiments, ELISAs were used to check the serum levels of **A** IL-6, **B** IL-1 and **C** TNF-α. A biochemical analyzer was used to check the blood levels of **D** glucose, **E** triglycerides and **F** total cholesterol. Data were plotted as the means ± SEM. *P < 0.05, **P < 0.01, ***P < 0.001. Control = animals treated with the vehicle (0.9% NaCl; n = 6); other three groups (n = 8 for each)

### L-carnitine regulates the cell metabolism by reducing the elevated serum IL-6 and IL-1 levels, and affecting glycolipid metabolism

Pro-inflammatory cytokines play key roles in the progression of cancer cachexia [33]. Cytokine measurements showed that the serum levels of IL-6, IL-1 and TNF-ɑ were all elevated in CT26 tumor-bearing mice compared with normal control mice (Fig. 6A–C). Treatment with 10 mg/kg/d of L-carnitine significantly decreased the serum levels of IL-6 and IL-1 compared with the cachexia control, but did not decrease the serum level of TNF-ɑ (Fig. 6C).

The blood glucose level was significantly decreased in cachectic mice compared to vehicle controls, and this phenomenon was exacerbated by treatment with 1 mg/kg/d or 10 mg/kg/d of L-carnitine (Fig. 6D).

**Fig. 7:**
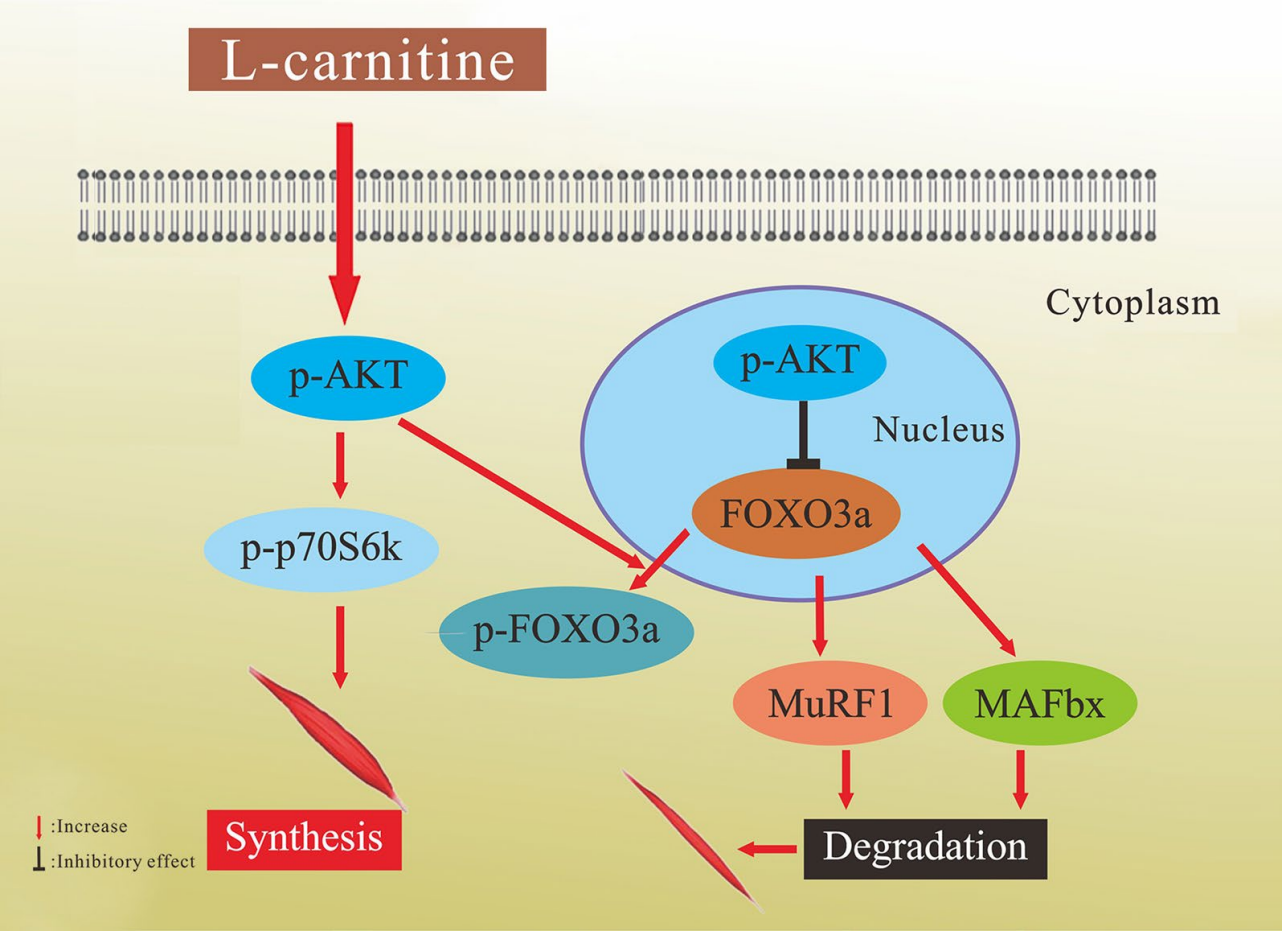
The hypothesized mechanism underlying the protective effects of L-carnitine against protein wasting

Compared to vehicle controls, the serum levels of total triglycerides and cholesterol were increased in cachectic mice, but the increase was not significant. However, the serum levels of total triglycerides and cholesterol were significantly increased in mice treated with either dose of L-carnitine, with increases over 100% (Fig. 6E) and nearly 50% (Fig. 6F) compared with cachectic mice and vehicle control for the 10 mg/kg and 1 mg/kg doses, respectively. These results suggested L-carnitine through the AKT/FOXO3a/MaFbx axis and p70S6K (Fig. 7).

## Discussion

The present studies demonstrate that L-carnitine ameliorates the muscle wasting of cancer cachexia in in vivo and in vitro models. These effects may result from an improved inflammatory status, inhibition of the muscle catabolic pathways and/or stimulation of the anabolic pathways.

Cancer cachexia not only negatively affects the quality of life of patients, but is also associated with a reduced efficacy and increased toxicity of chemotherapy, thereby contributing to mortality [34]. Body weight loss, muscle wasting, anorexia and inflammation are the four key characteristics of cancer cachexia. The outcomes of patients with cancer cachexia are currently poor. Specific interventions preventing or reversing cachexia are anticipated to have an important positive impact on the overall tumor disease outcome [34]. Pharmaceutical agents such as Cox-2 inhibitors, thalidomide, megestrol, medroxyprogesterone acetate, melatonin, and DCA, and supplemental nutrients or antioxidants from food [35], such as ω-3 poly-unsaturated fatty acids, vitamin C, vitamin D, vitamin E, β-hydroxy-β-methylbutyrate (HMB) [36] and lycopene, represent potential anti-cachexia treatments that are being explored in clinical trials.

L-carnitine, a conditional essential amino acid, is deficient in patients and animals with cancer cachexia [37, 38]. L-Carnitine supplementation led to an increase of body mass index and an increase in overall survival in cancer patients [35]. In humans, L-carnitine is part of the active component of carnitine palmityl transferase I and II (CPT I–II) [39], and facilitates the transfer of acyl groups at the interface between fatty acid and carbohydrate metabolism, promoting fatty acid oxidation and nonoxidative glucose disposal. A study by Seelaender et al. showed that CPT II activity is decreased in the liver mitochondria of tumor-bearing cachectic rats [33]. Two other studies showed that L-carnitine attenuates cancer cachexia through regulating the activity of CPT [24, 38]. However, the anti-cachexia effects of L-carnitine might be more complex.

There are various forms of carnitine in patient’s serum, including long-chain acylcarnitine, short-chain acylcarnitine, and free carnitine. All of the forms of carnitine were lower in cachectic patients with both gastrointestinal (GI) cancer and non-gastrointestinal (non-GI) cancer compared with healthy subjects [21]. The patients with a BMI ≤ 19 kg/m^2^ had lower serum free L-carnitine and total L-carnitine levels than those with a BMI > 19 kg/m^2^ [40]. These low serum carnitine levels in cachectic patients contribute to the progression of the condition in cancer patients [21]. Patients with cancer cachexia also had significant deficiencies of L-carnitine in their skeletal muscles.

Although providing supplemental L-carnitine or increasing the CPT levels is considered to be beneficial to ameliorate cancer cachexia, the precise mechanism is not fully understood. Our present study implies that L-carnitine directly ameliorates cancer-induced cachexic muscle atrophy in vivo and in vitro, and these effects result from the improved inflammatory status, inhibition of the muscle catabolic pathways and stimulation of the anabolic pathways.

Anorexia is common in cachexic cancer patients [41]. Anorexia reduces the intake of various nutrients, leading to further metabolic disorders. Gramignano et al. previously showed that L-carnitine treatment efficiently improved appetite and increased the lean body mass in patients with advanced cancer [42]. Our present study demonstrated that CT26 tumor-bearing mice had decreased food intake from D10 until the end of the experiments, and even high-dose L-carnitine did not improve the anorexia within this time period (Fig. 1H). This was in disagreement with the findings reported by Busquets et al. [43] and of another previous study, which showed that L-carnitine could ameliorate the anorexia of rats with cancer cachexia. The differences may be due to the differences in experimental animal species or in experimental differences.

Numerous cell signaling pathways are involved in the initiation and progression of cancer cachexia, including the TNF-ɑ pathway, TGFβ/p38/MAPK pathway, NFκB pathway, IL-6 pathway, IGF/Akt pathway and FOXO1/FOXO3a pathway, among others [44]. Within these pathways, many cell cytokines, including those associated with inflammation, such as IL-1, IL-6, TNF-10, IL-22, and TNF-alpha, contribute to the anorexia of cancer patients [8, 9, 12].

An increasing number of studies are showing that TNF-α and IL-6 are the primary inflammatory cytokines implicated in cancer cachexia [7, 9], and these have emerged as critical factors related to the loss of muscle mass. Increased levels of IL-6 are associated with significantly greater weight loss and a poorer overall prognosis [13]. In our study, CT26 tumor-bearing mice exhibited weight loss and significantly elevated serum IL-6 and IL-1 levels. L-carnitine treatment decreased the IL-6 level to that of the non-tumor-bearing mice. The impact of L-carnitine treatment on the TNF-ɑ level is difficult to determine [45]. Our study did not find a significant reduction in the TNF-ɑ level. This is in agreement with another study that showed that L-carnitine had a more limited effect on TNF-α than on IL-6 in the same experimental model after seven days of carnitine intervention [38]. In our experiments, we measured the expression of inflammatory cytokines after 18 days of L-carnitine intervention. It is possible that the different time points of detection resulted in the different results among the various studies. Although the TNF-α-initiated cachexic changes in C2C12 cells comprise a classic cell model of cancer cachexia, we consider that TNF-alpha is certainly not the only cytokine that is important for cancer cachexia. Multi-disciplinary treatment may be required to improve cancer cachexia, including the use of oral nutritional supplements, L-carnitine, thalidomide, n-3 fatty acids, and megestrol acetate, as was shown in a five-arm randomized clinical trial [46]. A future study with frequent blood collection (i.e., in a rat model to permit the collection of larger volumes of blood at more frequent intervals) would be useful to better understand the temporal changes in the expression of the various cytokines, and their correlation with both the pathological and behavioral changes in the animals.

Cancer cachexia induces major metabolic disruptions, including alterations in lipid metabolism and glycometabolism, that contribute to the aggravation of cachexia symptoms [45]. Our data showed that the blood glucose level decreased significantly in the CT26 tumor-bearing mice, which may have been due to the Warburg effect [47]. High and unstable blood glucose levels will promote tumor cell growth [48], so a reduction in glucose following L-carnitine treatment might have been beneficial to inhibit tumor growth in the CT26 tumor-bearing mice. In cachexic subjects, sustaining catabolism and inflammation will accelerate the production of TNF-ɑ, IL-6 and IL-1 to block lipoprotein lipase [49] and the L-carnitine-induced fatty acid transfer system, leading to hypertriglyceridemia and hypercholesterolemia [21, 22]. Our data showed that the serum levels of triglycerides and total cholesterol increased in the CT26 tumor-bearing mice compared with normal controls, but the increase was not significant. L-carnitine treatment could not reverse the hypertriglyceridemia and hypercholesterolemia in the cachexic mice, and actually increased the serum levels of triglycerides and total cholesterol. Our data were consistent with those of a single-center randomized control trial [50], which showed that oral L-carnitine supplementation for 6 months significantly increased the serum levels of low-density lipoprotein cholesterol (LDL-C) and triglycerides in patients on hemodialysis. Since the main function of L-carnitine is to promote the β-oxidation of long-chain fatty acids, this activity may increase lipocatabolic metabolism, thus increasing the cholesterol and triglyceride levels. We will explore the precise mechanism(s) underlying these findings in a future study.

Although L-carnitine could attenuate the symptoms of cancer cachexia [51], the precise mechanisms underlying these effects is still unknown. Muscle wasting is the key pathophysiological process underlying cancer cachexia. For the last two decades, skeletal muscle was the main target of therapy [40], and drug discovery programs focused on strategies to inhibit the muscle catabolic pathways and stimulate the anabolic pathways [52]. Protein synthesis and degradation need be in balance to keep the appropriate muscle mass [53]. A negative balance occurs when protein degradation is greater than protein synthesis. With regard to muscle atrophy in cachexia, studies have focused on different cell signaling pathways, including the myostatin pathway, TNF-ɑ pathway, TGFβ/p38/MAPK pathway, NFκB pathway, IL-6 pathway [12], Notch/β-catenin pathway, corticosteroid pathway, IGF/Akt pathway [54], and FOXO1 /FOXO3a pathway [55], among others. The activation of the FOXO family, which may be due to TNF-α, soluble TNF-like weak inducer of apoptosis (TWEAK), or IL-1, is common in skeletal muscle atrophy [15, 16]. FOXO3a induces a set of atrophy-related genes, specifically the muscle-specific ubiquitin ligases, MAFbx and MuRF-1, which promote the breakdown of the myofibrillar apparatus [17]. FOXO3 activation has been shown to induce MAFbx and MuRF1 transcription, stimulate catabolism and cause muscle wasting [55, 56]. FOXO1 transgenic mice have a lower skeletal muscle mass than non-transgenic mice [57]. Previous studies showed that overexpression of FOXO1/3a in muscle was associated with remarkable decreases in myotube diameter and fiber size in mice [57]. A study by Zhou et al. study suggested that the reversal of muscle wasting in cancer cachexia leads to prolonged survival [58].

Our data support a role for FOXO in atrophy, which is consistent with the studies mentioned above [16, 57]. Myotubes could be induced to hypertrophy by the PI3K/Akt pathway, which increases protein synthesis and blocks the up-regulation of MAFbx and MuRF1 that occurs during muscle atrophy [54, 59].

PI3K/AKT signaling leads to the activation of mTOR, p70S6, and various other pathways, which also promote muscle synthesis [60]. Our study showed that L-carnitine reverses the TNF-α-induced muscle cell atrophy caused by regulation of the Akt/P70S6K/FOXO3a pathways, and by inhibiting muscle-specific ubiquitin ligases. A study by Peng et al. showed that disruption of the Akt1 and Akt2 genes in mice led to significant muscle atrophy [61]. Studies have shown that Akt phosphorylation inhibits FOXO3a and blocks the upregulation of MuRF1 and MAFbx during muscle atrophy [16, 54]. Therefore, we examined the effects of an inhibitor of AKT1 (siRNA) in the cancer cachexia models, and found that the expression of the Akt/FOXO3a signaling pathways had changed, with higher expression of MaFbx. When the various groups were treated with L-carnitine, the expression levels of p-p70S6, p70S6, p-FOXO3a, FOXO3a, Akt, p-Akt, MuRF1 and MaFbx were all changed. Our results strongly suggest that the muscle atrophy induced by TNF-α is regulated, at least in part, by the Akt inhibition associated with FOXO3a and MaFbx. In the present study, L-carnitine increased the level of phosphorylated Akt, which may have been the reason why the differentiated muscle cells’ atrophy was improved.

Our current studies showed that L-carnitine efficiently ameliorated ongoing muscle atrophy in a cell cachexia model and murine cancer cachexia model. It should be noted that L-carnitine enhanced p-AKT, p-P70S6, and p-FOXO3a expression and inhibited the expression of MuRF1 and MaFbx in the gastrocnemius muscle, with major decreases in inflammatory cytokines IL-6 and IL-1.

## Conclusion

Cachexic patients usually present with rapid weight loss, extreme muscle loss, anorexia, and ultimately, multiple organ dysfunction syndromes. Our data showed that L-carnitine could ameliorate muscle wasting in in vitro and in vivo models of cachexia. L-carnitine treatment also ameliorated inflammation in the tumor-bearing mouse model, which suggests that it could be beneficial for cancer patients. Further, L-carnitine decreased skeletal muscle wasting, possibly by blocking ubiquitin-mediated protein degradation via the Akt/FOXO3/MaFbx axis. Further studies will be needed to confirm whether L-carnitine is effective in the clinical setting.

## Data Availability

The data used to support the findings of this study are available from the corresponding author upon request.

## Abbreviations

AKT: Protein kinase B
BMI: Body mass index
CFA: Cross-sectional fiber area
Cox-2: Cytochrome c oxidase subunit II
CPTI- II: Carnitine palmityl transferase I and II
FOXO: Forkhead box O transcription factor family
FOXO3a: Forkhead box O transcription factor 3a
GI: Gastrointestinal
GM: Gastrocnemius muscle
HE: Hematoxylin and eosin
HMB: β-Hydroxy-β-methylbutyrate
IGF1: Insulin like growth factor 1
IL-1: Interleukin-1
IL-6: Interleukin-6
LC: L-carnitine
MaFbx: Muscle atrophy F-box protein
MAPK: Mitogen-activated protein kinase
mTOR: Mammalian target of rapamycin
MuRF1: Muscle RING-type E3 ubiquitin ligase 1
NF-κB: Nuclear factor NF-kappa-B
PIF: Proteolysis-inducing factor
TGFβ: Transforming growth factor beta
TNF-α: Tumor Necrosis Factor α
TWEAK: TNF-like weak inducer of apoptosis
